# Intra-arterial lidocaine as a pain management strategy for uterine artery embolisation: a systematic review and meta-analysis

**DOI:** 10.1186/s42155-026-00771-y

**Published:** 2026-09-16

**Authors:** Tarun Haridasan, Abena Kena, Larry Nguyen, Kiat Tsong Tan

**Affiliations:** 1 Michael G. DeGroote School of Medicine, McMaster University, 1280 Main Street West, Hamilton, ON L8S 4L8 Canada; 2 Department of Medical Imaging, Niagara Health System, St. Catharines, ON L2S 0A9 Canada

## Abstract

Post-procedural pain remains a significant challenge following uterine artery embolisation (UAE) and intra-arterial lidocaine (IAL) has emerged as a potential adjunctive analgesic. This systematic review and meta-analysis evaluated the efficacy and safety of IAL during UAE. MEDLINE, Embase, and the Cochrane Library were searched from inception to June 2026, identifying eight studies including 692 patients. IAL significantly reduced pain at 4 h post-procedure and lowered the maximum pain experienced within the first 24 h. However, there were no significant differences in pain immediately after the procedure, at 7 or 24 h, opioid consumption, or inflammatory markers. Serious adverse events were uncommon. These findings suggest that IAL provides meaningful early pain relief and may serve as a safe adjunct in pain management for UAE.

**Systematic review registration** PROSPERO CRD420261428710

**Supplementary Information:**

The online version contains supplementary material available at https://doi.org/10.1186/s42155-026-00771-y.

## Background

Uterine fibroids, also known as leiomyomas, are benign monoclonal growths that emerge from the myometrium and fibroblasts [1, 2]. Fibroids are one of the most common gynaecological tumours, affecting 70 to 80% of women over the age of 50 [2]. The exact aetiology of fibroids is unknown, but is linked to various risk factors, including obesity, hypertension, early menarche, nulliparity, older age, and a family history of fibroids [3]. Fibroids are also more common, larger, greater in number, and present at a younger age in women of African ancestry compared to White or Asian women [4]. Although most women with Uterine fibroids are asymptomatic and are discovered incidentally on clinical examination or imaging, around 30% of patients will experience severe symptoms, such as uterine bleeding, anaemia, back pain, constipation, and/or infertility, which require intervention [2, 3].

Surgical treatments, including hysterectomy, have been the conventional choice for managing fibroids [5]. Uterine artery embolisation (UAE) has emerged as an effective, minimally invasive alternative for patients seeking preservation of the uterus and fertility [6]. UAE works by occluding the blood supply to the uterus through administration of embolic agents to the uterine artery, inducing infarction of the fibroids [6]. Although UAE is effective in reducing the fibroid volume, post-procedure pain remains a challenge.

Despite these advantages, post-procedure pain remains one of the most significant challenges associated with UAE, with patients experiencing intense pain within a few hours following the procedure [7, 8]. Several pain management strategies exist for UAE, including opioid regimens, nonsteroidal anti-inflammatory drugs (NSAIDs), and superior hypogastric nerve blocks (SHNB) [8, 9]. Among analgesic strategies, intra-arterial lidocaine (IAL) has gained increasing interest where lidocaine is administered directly through the microcatheter to the uterine arterial circulation either during or immediately following embolisation [8, 10]. Although several studies have evaluated the use of IAL during UAE, the outcomes have been inconsistent. Some studies have documented adverse reactions such as neurological symptoms, arrhythmias, and less commonly, seizures [11]. Some studies have reported minimal improvements in pain control and opioid consumption [8]. In addition, concerns have been raised that lidocaine may induce uterine artery vasospasm, potentially affecting embolic agent delivery and the success of the intervention [10].

At present, the efficacy of lidocaine in pain relief after UAE remains controversial [12]. Therefore, an updated systematic review and meta-analysis was conducted to evaluate the efficacy and safety of IAL as a pain management strategy for UAE. This review aims to synthesise the available evidence on post-procedural pain, analgesic consumption, and the adverse effects of IAL for UAE.

## Methods

### Study design and reporting

This systematic review and meta-analysis was conducted in accordance with the Preferred Reporting Items for Systematic Reviews and Meta-Analyses (PRISMA) 2020 guidelines [13]. This review was registered in the International Prospective Register of Systematic Reviews (PROSPERO; registration number CRD420261428710).

### Search strategy and data source

A literature search was conducted in MEDLINE (Ovid), Embase (Ovid), and Cochrane Library from database inception to June 19, 2026. The search strategy was developed using a combination of MeSH terms and keywords related to uterine artery embolization and lidocaine. The MeSH terms used in this search included uterine artery embolization and lidocaine, as well as relevant free-text keywords, including uterine fibroid embolization, intra-arterial lidocaine, lignocaine, and more. The exact search strategy for each database is available in the review protocol, which has been publicly registered with PROSPERO. Reference lists of included studies were also hand-searched for any additional articles.

### Eligibility criteria

Studies were considered eligible if they met the following criteria: (1) enrolled adult women undergoing uterine artery embolisation (UAE); (2) evaluated intra-arterial lidocaine administered as the primary pain management strategy; (3) reported at least one prespecified outcome of interest; (4) were randomised controlled trials, prospective cohort studies, retrospective cohort studies, or case control studies; and (5) were published in English and available as full-text articles.

Studies were excluded if they (1) evaluated procedures other than UAE; (2) did not investigate intra-arterial lidocaine administration; (3) were animal, cadaveric, or preclinical studies; (4) were review articles, systematic reviews, meta-analyses, editorials, letters, commentaries, expert opinions, or conference abstracts without a corresponding full-text publication.

### Selection process and data extraction

All identified studies were imported into Covidence for removal of duplicates and screening. Two reviewers from the team (T.H., A.K., L.N.) independently screened each study for eligibility at both title and abstract and full-text stages. Data were also extracted independently by two reviewers (T.H., A.K., L.N.) using a standardised data extraction form on Covidence. Disagreements at all stages were resolved through discussion with a third reviewer until consensus was reached.

### Outcomes

The primary outcome was post-procedure pain severity, measured using the Visual Analog Scale (VAS) and Numeric Rating Scale (NRS). Secondary outcomes included opioid consumption, length of hospital stay, time to discharge, patient satisfaction, technical success, and procedure-related complications and readmissions. Where studies reported pain at multiple time points, data were extracted separately for each reported time point.

### Risk of bias assessment

Risk of bias was assessed independently by two reviewers (T.H., A.K., L.N.). Randomised controlled trials were assessed using the Cochrane Risk of Bias 2 (RoB 2) tool [14]. Non-randomised studies were assessed using the Risk of Bias in Non-Randomised Studies of Interventions (ROBINS-I) tool [14].

### Synthesis and statistical analysis

Meta-analyses were performed using Review Manager (RevMan, version 5.4). Continuous outcomes were pooled using mean differences while dichotomous outcomes were pooled using odds ratios. Random-effects models were used for all meta-analyses, with the inverse variance method for continuous outcomes and the Mantel–Haenszel method for dichotomous outcomes. Statistical heterogeneity was assessed using the I^2^ statistic. When studies reported continuous outcomes as medians with interquartile ranges or other non-standard forms, means and standard deviations were estimated using established methods where appropriate [15]. Where quantitative synthesis was not appropriate due to heterogeneity or insufficient data, findings were summarized narratively. In Cai et al. [11], which had both unilateral and bilateral IAL administration arms, only the bilateral arm was included in the primary meta-analysis to maintain consistency with the intervention used in the remaining studies. In Noel-Lamy et al. [16], the two intervention groups, post-UAE administration and during UAE administration, were combined into one intervention group using recommended methods from the Cochrane Handbook for Systematic Reviews of Interventions [17].

## Results

### Study selection

**Fig. 1:**
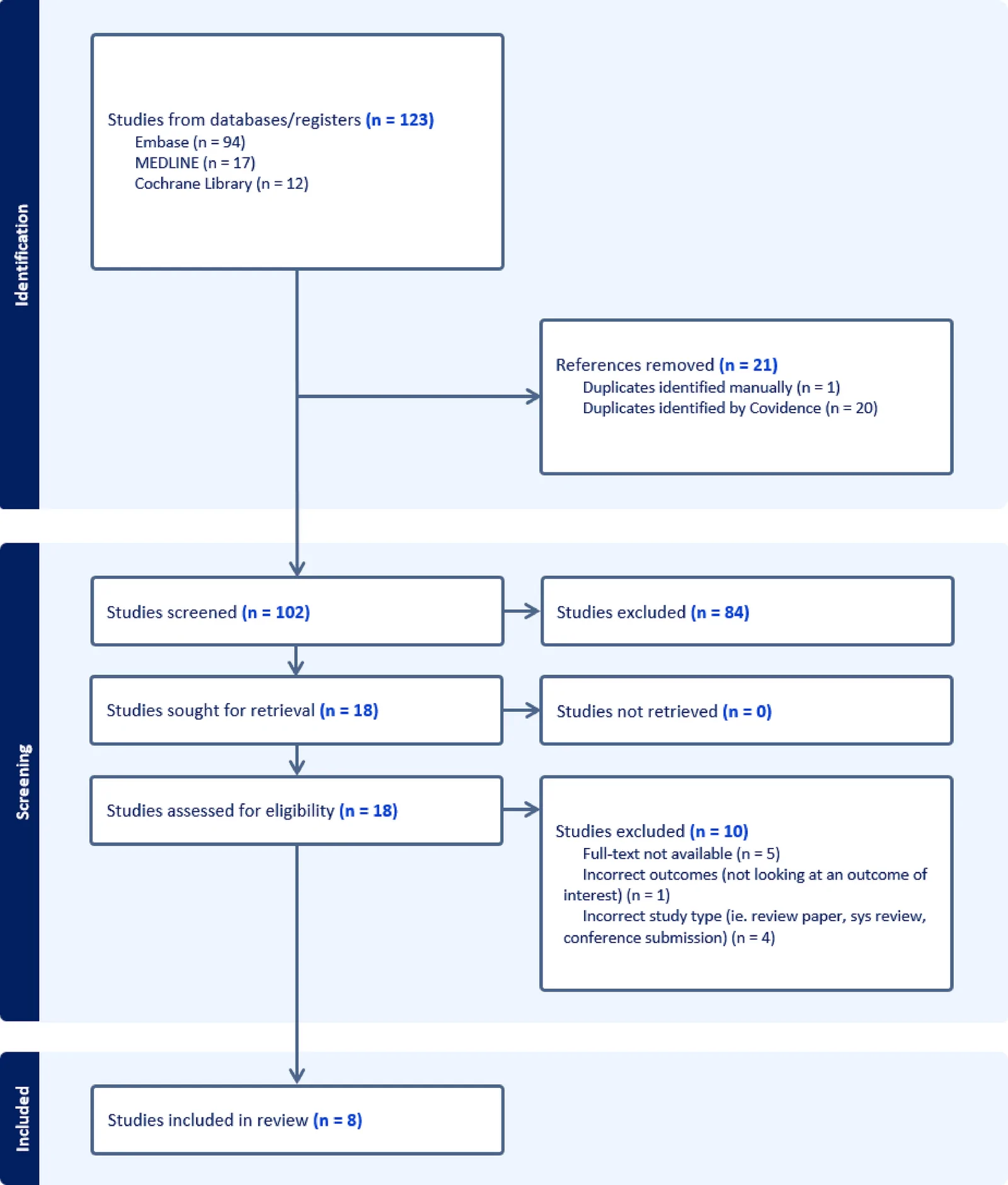
PRISMA flow diagram of study selection process

The literature search identified 123 records from the databases. After removal of 21 duplicate records, 102 studies underwent title and abstract screening, of which 84 were excluded. 18 full-text articles were assessed for eligibility, of which 10 studies were excluded because the full text was unavailable (n = 5), the study did not report an outcome of interest (n = 1), or the publication was an ineligible study type (n = 4). Eight studies met the eligibility criteria and were included in the systematic review (Fig. 1).

### Study characteristics

A total of eight studies published between 2001 and 2025 were included in the systematic review comprising a total of 692 patients. Four studies were randomised controlled trials, while the remaining four were retrospective cohort studies. The studies were conducted across five countries, including China (n = 3), the USA (n = 2), Canada (n = 1), Japan (n = 1), and the Republic of Korea (n = 1). The studies evaluated the use of IAL during UAE for the treatment symptomatic uterine fibroids (n = 7) and caesarean scar pregnancy (n = 1). Post-procedural pain was assessed using a visual analogue scale in six studies, a numerical rating scale in one study, and a six-level ordinal pain scale in one study. Table 1 summarizes relevant study characteristics from all included studies. Table 2 describes relevant patient characteristics from all included studies. Table 3 describes relevant intervention characteristics from all included studies.

**Table 1:** Study Characteristics of Included Studies. Characteristics of the studies include country of origin, study design, indication for uterine artery embolization (UAE), pain assessment scale, and number of patients enrolled. UAE, uterine artery embolization; VAS, visual analog scale; NRS, numeric rating scale; RCT, randomized controlled trial

Study, Year	Country	Design	Indications for UAE	Pain Scale	Patients
Alqahtani 2024 [18]	Korea	Retrospective	Uterine fibroids	VAS	46
Cai 2025 [11]	China	Retrospective	Cesarean scar pregnancy	VAS	255
Duvnjak 2020 [19]	United States	RCT	Uterine fibroids	VAS	36
Katsumori 2020 [20]	Japan	Retrospective	Uterine fibroids	VAS	100
Keyoung 2001 [10]	United States	RCT	Uterine fibroids	NRS	18
Noel-Lamy 2017 [16]	Canada	RCT	Uterine fibroids	VAS (100-pt)	60
Tang 2022 [12]	China	Retrospective	Uterine fibroids	VAS	131
Zhan 2005 [21]	China	RCT	Uterine fibroids	6-level ordinal pain scale: none, little, slight, apparent, severe, very severe	46

**Table 2:** Patient Characteristics of Included Studies. Baseline characteristics of patients included in each study. Reported variables include treatment group, number of patients, mean age, body mass index (BMI), uterine volume, and dominant fibroid volume. IAL, intra-arterial lidocaine; BMI, body mass index; NR, not reported

Study	Procedure	Patients	Mean Age	Mean BMI	Uterine Volume (cm^3^)	Dominant Fibroid Volume (cm^3^)
Alqahtani 2024 [18]	IAL	23	44.2 ± 5 s	22.5 ± 2.5	384.6 ± 218.8	108 ± 84.7
	Control	23	43.9 ± 4	22 ± 2.7	410.9 ± 207.1	119.1 ± 81.6
Cai 2025 [11]	IAL (Unilateral)	39	33.5 ± 3.1	22.9 ± 2.6	NR	NR
	IAL	32	33.4 ± 3.2	23.4 ± 2.5	NR	NR
	Control	184	33.7 ± 3.6	23.2 ± 2.2	NR	NR
Duvnjak 2020 [19]	IAL	20	44.4 ± 3.3	NR	NR	183.1 ± 206.5
	Control	16	43.1 ± 5.11	NR	NR	262.8 ± 188.7
Katsumori 2020 [20]	IAL	50	43.5 ± 4.3	21.9 ± 2.9	1,050 ± 530	425 ± 406
	Control	50	42.9 ± 5.0	23.1 ± 4.7	1,040 ± 639	426 ± 411
Keyoung 2001 [10]	IAL	10	NR	NR	525.5 (238.3–812.7)	130.4 (59.8–201.0)
	Control	8	NR	NR	467.6 (345.4–589.8)	173.5 (97.6–249.9)
Noel-Lamy 2017 [16]	IAL (post)	20	44.8 ± 6.8	NR	912.5 ± 809.2	453.2 ± 495.1
	IAL (during)	20	47.1 ± 6.1	NR	658.1 ± 501.6	245.2 ± 205.8
	Control	20	48.4 ± 3.6	NR	636.8 ± 505.4	214.7 ± 257
Tang 2022 [12]	IAL	36	44.2 ± 3.6	22.4 ± 2.6	562.5 (258.3—953.1)	163.8 (103.3—331.5)
	Control	45	45.4 ± 3.2	23.2 ± 3.8	268.3 (201.0–1095.6)	67.3 (51.6–258.6)
Zhan 2005 [21]	IAL	23	41	NR	NR	NR
	Control	23		NR	NR	NR

**Table 3:** Intervention Characteristics of Included Studies. Details of the intra-arterial lidocaine (IAL) intervention and uterine artery embolization (UAE) procedures across included studies, including lidocaine concentration, dose, volume, timing of administration relative to embolization, and embolic agent used. IAL, intra-arterial lidocaine; UAE, uterine artery embolization

Study, Year	Lidocaine Concentration	Lidocaine Dose (mg)	Lidocaine Volume (mL)	Timing of Lidocaine Administration	Embolic Agent Used for UAE
Alqahtani 2024 [18]	1.00%	100	10	Immediately after embolization	Polyvinyl alcohol particles; 355–700 µm
Cai 2025 [11]	2.00%	80	4	Before embolization	Gelatin sponge; 560–710 μm
Duvnjak 2020 [19]	1.00%	200	20	Immediately after embolization	Tris-acryl gelatin; 500–900 μm
Katsumori 2020 [20]	1.00%	80	8	Immediately after embolization	Tris-acryl gelatin; 500–900 μm
Keyoung 2001 [10]	1.00%	200	20	Immediately before embolization	Polyvinyl alcohol; 500–710 μm
Noel-Lamy 2017 [16]	1.00%	100	20	Either during UAE mixed with microspheres or immediately after embolization	Nonspherical polyvinyl alcohol; 300–700 µm
Tang 2022 [12]	0.50%	100	20	During UAE mixed with microspheres	Polyvinyl alcohol; 355–500 µm
Zhan 2005 [21]	0.67%	40	6	Immediately after embolization was complete	Polyvinyl alcohol; 500–710 µm; followed by 1–3 gelfoam pledgets

### Pain scores

**Fig. 2:**
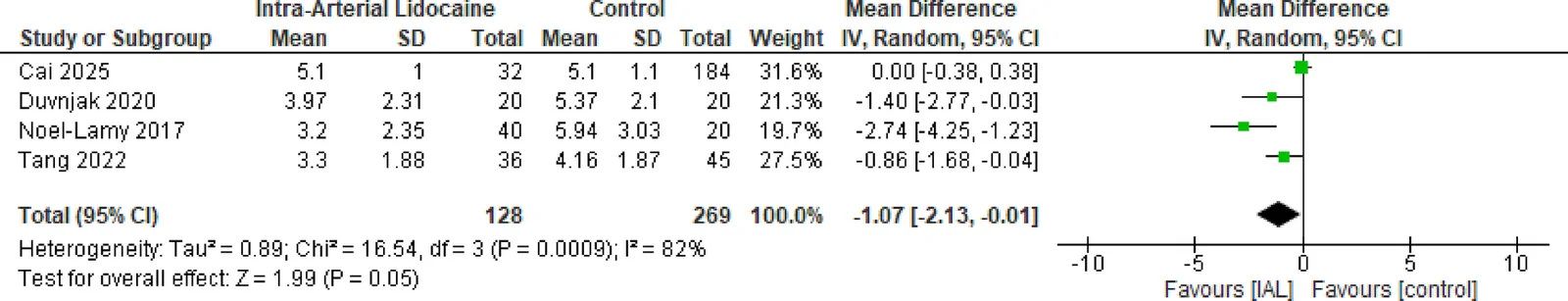
Forest plot comparing 4-h post-procedure pain scores for intra-arterial lidocaine (IAL) and control. M-H, Mantel–Haenszel; IV, inverse variance; CI, confidence interval

Since postoperative pain score was reported at different time points across the studies, meta-analysis could only be performed for the time points with sufficient data, which were at 0, 4, 7, and 24 h following the procedure. At 4 h post-procedure, IAL was associated with significantly lower pain scores than control (four studies, MD = −1.07; 95% CI −2.13 to −0.01; P = 0.05; I^2^ = 82%; Fig. 2). No significant differences were observed at 0 h (three studies, MD = −0.54; 95% CI −2.03 to 0.96; p = 0.48; I^2^ = 96%), 7 h (three studies, 177 patients; MD = −0.36; 95% CI −0.98 to 0.26; P = 0.26; I^2^ = 0%), or 24 h (four studies, 393 patients; MD = − 0.09; 95% CI −0.52 to 0.34; P = 0.70; I^2^ = 24%). Forest plots for the remaining analyses are provided in the Supplementary information (Supplementary Figs. S1–S4).

**Fig. 3:**
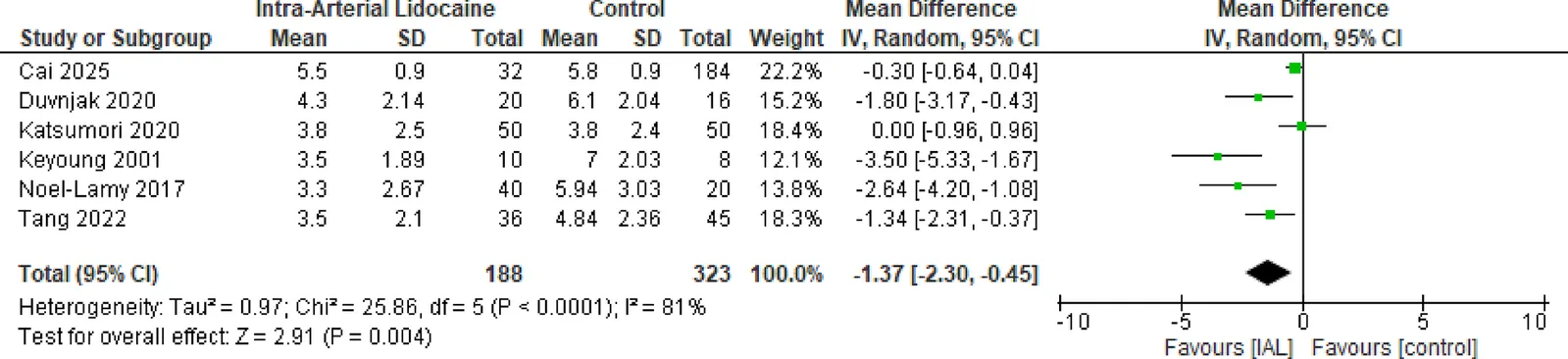
Forest plot comparing maximum 24-h pain scores for intra-arterial lidocaine (IAL) and control. M–H, Mantel–Haenszel; IV, inverse variance; CI, confidence interval

A total of six studies (n = 511 patients) were included in the meta-analysis for maximum 24-h pain score (Fig. 3). Since not all studies directly reported the maximum 24-h pain score, the maximum was obtained by selecting the highest pain score across the reported time points. The 24-h pain score represents the pain score measured specifically at the 24-h time point, while the maximum 24-h pain score represents the highest pain score at any time point during the first 24 h following the procedure. IAL significantly reduced the maximum 24-h pain compared to control (MD = −1.37; 95% CI −2.30 to −0.45; p = 0.004), although substantial heterogeneity was observed (I^2^ = 81%).

### Opioid consumption

**Fig. 4:**
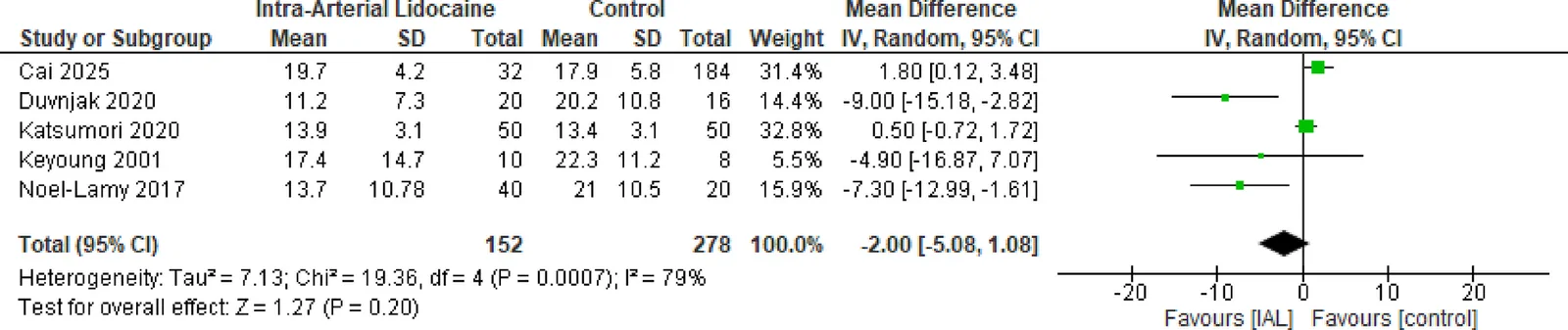
Forest plot comparing 24-h morphine consumption for intra-arterial lidocaine (IAL) and control. M-H, Mantel–Haenszel; IV, inverse variance; CI, confidence interval

Five studies (n = 430 patients) were included in the meta-analysis of 24-h morphine consumption (Fig. 4). No significant difference was observed between the IAL and control group (MD = −2.00 mg; 95% CI − 5.08 to 1.08; P = 0.20; I^2^ = 79%). Two additional studies used different opioids, which were not included in the quantitative synthesis. Alqahtani et al. [18] reported higher 24-h fentanyl consumption in the IAL group compared to control (1037 ± 573 µg vs. 881 ± 419 µg), while Tang et al. [12] reported lower 24-h sufentanil consumption in the IAL group than the control (38.3 ± 6.25 mg vs. 45.7 ± 6.51 mg).

### Inflammatory markers

Two studies (n = 127 patients) reported 24-h CRP and WBC counts. No significant difference was observed in the CRP (MD = − 5.58; 95% CI − 14.79 to 3.63; p = 0.23; I^2^ = 89%) and WBC (MD = − 1.14; 95% CI − 2.61 to 0.32; p = 0.13; I^2^ = 43%) between the groups (Supplementary Figs. S5 and S6).

### Sensitivity analysis

Since significant heterogeneity was present in some of the meta-analyses, a sensitivity analysis was performed by excluding the study involving caesarean scar pregnancy [11], as this is a clinically distinct patient population from the remaining studies which perform UAE for fibroids. Following its exclusion, the heterogeneity was greatly reduced in all analyses while the pooled effect size remained the same. For the 4-h pain score, exclusion of this study strengthened the pooled treatment effect (MD −1.52, 95% CI −2.58 to −0.46; p = 0.005) and reduced heterogeneity from I^2^ = 82% to 56% (Supplementary Fig. S7). For maximum 24-h pain scores, the pooled effect also became slightly larger (MD −1.71; 95% CI −2.84 to −0.57; p = 0.003), while heterogeneity decreased modestly from I^2^ = 81% to 75% (Supplementary Fig. S8).

### Risk of bias

**Fig. 5:**
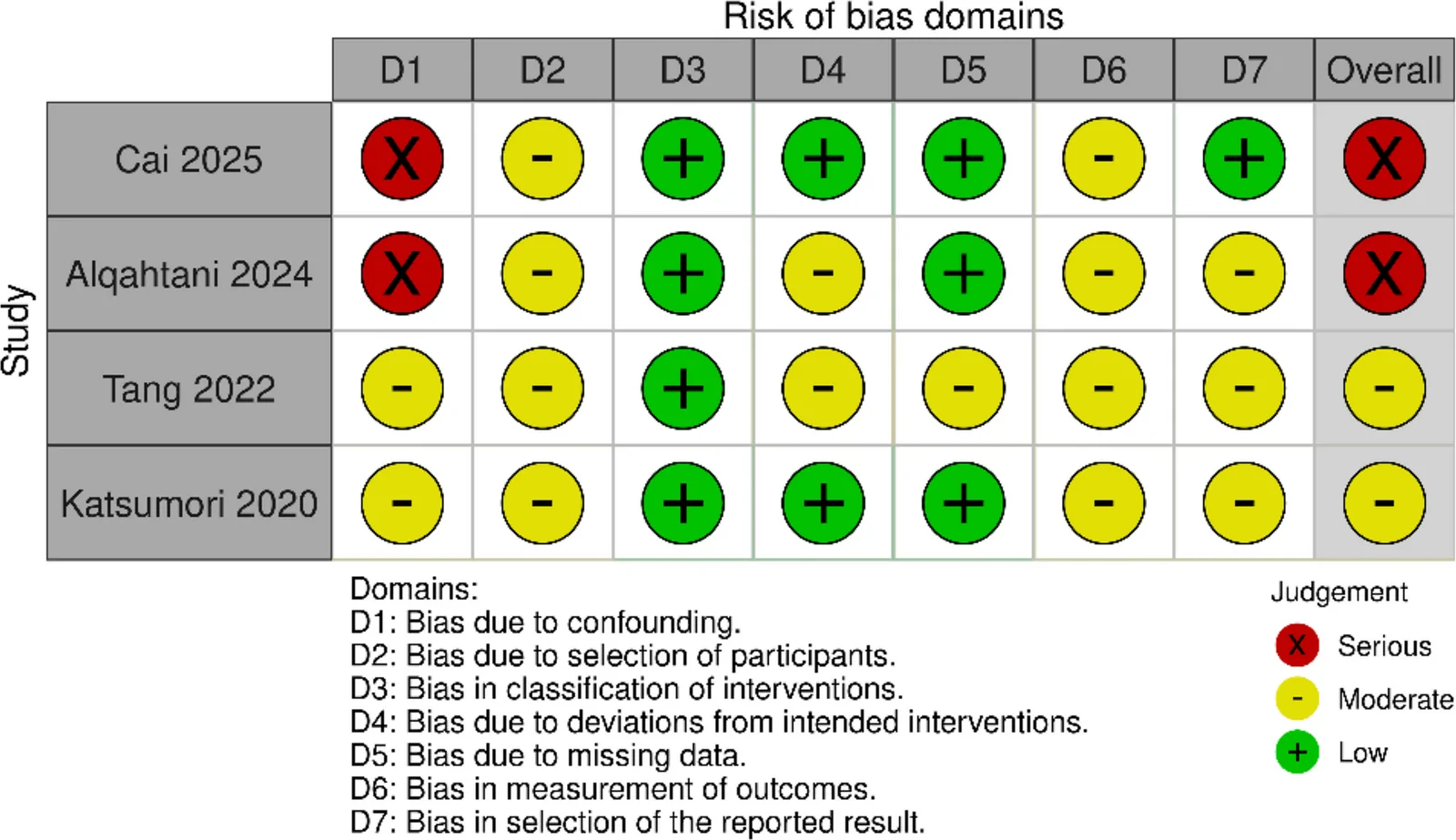
ROBINS-I risk of bias for non-randomised studies. D1, bias due to confounding; D2, bias in selection of participants; D3, bias in classification of interventions; D4, bias due to deviations from intended interventions; D5, bias due to missing data; D6, bias in measurement of outcomes; and D7, bias in selection of the reported result. Green (+) indicates low risk of bias, yellow (-) indicates moderate risk, and red (X) indicates serious risk

**Fig. 6:**
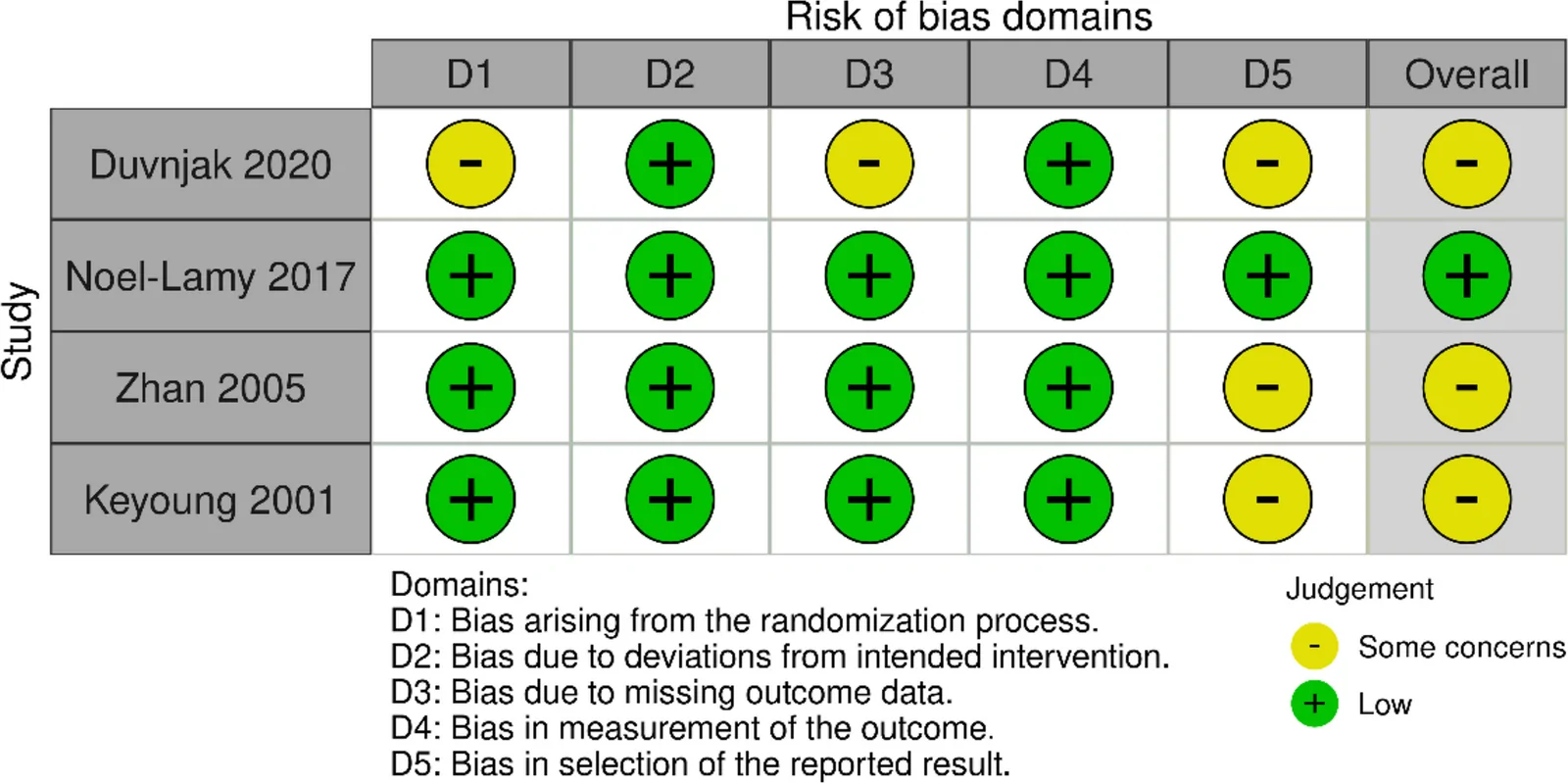
Cochrane RoB 2 assessments for randomised studies. D1, bias arising from the randomisation process; D2, bias due to deviations from intended interventions; D3, bias due to missing outcome data; D4, bias in measurement of the outcome; and D5, bias in selection of the reported result. Green (+) indicates low risk of bias, and yellow (-) indicates some concerns

Risk of bias was assessed using the Cochrane RoB 2 tool and ROBINS-I for randomised controlled trials and non-randomised studies, respectively. Among the four non-randomised studies, two studies [11, 18] were judged to have a serious risk of bias, while the other two studies [12, 20] had a moderate risk of bias. Amongst the four randomised studies, one study [16] was judged to be at low risk of bias across all domains, while the remaining three [10, 19, 21] had some minor concerns. The risk of bias for all studies, including the individual domains and overall judgement, is summarised in Figs. 5 and 6.

## Discussion

This systematic review and meta-analysis evaluated the use of IAL as pain management for UAE. Across eight studies, IAL was associated with significantly reduced pain scores at 4 h post-procedure and a reduction in the maximum pain experienced during the first 24 h post-procedure. However, no significant differences were observed in pain scores immediately after the procedure or at 7 and 24 h, nor was there a significant reduction in 24-h morphine consumption or inflammatory markers. Sensitivity analyses excluding Cai et al. [11], which involves caesarean scar pregnancy, reduced the statistical heterogeneity while maintaining the treatment effect.

Post-procedural pain is attributed to ischaemia from the embolisation of the uterine arteries. It is thought that lidocaine suppresses the pain response in the remaining myometrial branches that are preserved [8]. The observed reduction in pain at 4 h and in the maximum 24-h pain score is consistent with this proposed mechanism, as the greatest post-embolisation pain occurs within the first several hours after the procedure [8]. The absence of pain reduction at the later time points may reflect the short half-life of lidocaine [22, 23]. Although the pain scores were reduced at earlier time points, this did not translate to statistically significant lower opioid consumption. This may be due to lidocaine’s short duration of action, but also variation in the opioid protocols across institutions. Only the studies using morphine could be quantitatively synthesised, as fentanyl and sufentanil were used by one study each, limiting the available evidence.

Adverse events and complications were rare across the studies. Most studies reported no major procedure-related complications attributable to IAL. One study described transient bradycardia with loss of consciousness during lidocaine administration, which resolved following atropine administration [18]. Another study reported patients phoning back due to severe pain on day three after UAE, but there were no readmissions to hospital required [19]. The most notable adverse effects reported by Keyoung et al. [10] were severe vasospasms in 70% of patients, prompting early termination of the study. However, this was one of the first studies in the field from the early 2000 s, and subsequent studies did not reproduce these findings. Tang et al. [12] and Zhan et al. [21] reported mild vasospasm, but no severe complication. While the available evidence suggests that serious lidocaine-related complications are uncommon, firm conclusions regarding safety cannot be made due to the inconsistent reporting and limited sample sizes.

The present findings are mostly consistent with the broader literature and a previous systematic review conducted in this field. To our knowledge, only one systematic review exists on this topic. Liu and Li [24] also found substantially reduced pain scores at 4 h following the procedure, but no effect at later time points. The heterogeneity is also due to differences in the IAL protocols used across studies, as variation existed in lidocaine concentration (from 0.5 to 2%), dose (from 40 to 200 mg), and injection volume (from 4 to 20 mL), and embolic agent. Additionally, some studies administered lidocaine during the procedure along with the embolic agent, while others administered lidocaine immediately after embolisation. Because relatively few studies were available, subgroup analyses examining these procedural variables were not feasible. Future randomised controlled trials using standardised protocols are required to confirm the treatment effect.

The short duration of action observed with IAL also raises the idea that a different intra-arterial agent might be able to provide more sustained pain management. Ho and Tan [7] recently described the use of intra-arterial ropivacaine for pain management during UAE. Ropivacaine was selected because of its longer duration of action compared to lidocaine, and the patient experienced only mild pain throughout the procedure and early post-procedure period [7]. Although this evidence is currently limited to a single case report, it highlights the potential to use longer-acting agents to address one of the principal limitations of IAL identified in this review.

This study has several limitations that should be considered when interpreting the findings. First, there was moderate heterogeneity in terms of study design, patient population, and lidocaine administration protocols amongst the included studies. One included study also evaluated patients undergoing UAE for caesarean scar pregnancy rather than uterine fibroids, which is a clinically distinct population. Although sensitivity analysis demonstrated that exclusion of this study reduced heterogeneity while maintaining treatment effect, substantial heterogeneity remained for some outcomes. Another limitation of the study is the low sample size. Only eight studies with a total of 692 patients were available for inclusion, which limited the statistical power of the meta-analysis. Additionally, four of the eight included studies were retrospective cohort studies, two of which were judged to have a serious risk of bias. Therefore, the findings should be interpreted with caution, and larger randomised controlled trials using standardised IAL protocols and reporting scales are required to understand the role of IAL in UAE.

## Conclusion

In conclusion, our findings suggest that IAL provides meaningful improvements in early pain control, particularly at 4 h post-procedure and the maximum pain experienced within the first 24 h. However, there were no significant benefits for opioid consumption or inflammatory markers. The safety profile for IAL appears favourable, but more studies are required to conclude its technical simplicity, IAL can act as an adjunct to existing pain management protocols rather than a replacement for systemic analgesia. Future studies with larger sample sizes and standardised protocols are needed to better define its role in pain management for UAE.

## Supplementary Information

Supplementary Material 1: Supplementary Figure S1: Forest plot comparing 0-h post-procedure pain scores for intra-arterial lidocaine (IAL) and control. Supplementary Figure S2: Forest plot comparing 4-h post-procedure pain scores for intra-arterial lidocaine (IAL) and control. Supplementary Figure S3: Forest plot comparing 7-h post-procedure pain scores for intra-arterial lidocaine (IAL) and control. Supplementary Figure S4: Forest plot comparing 24-h post-procedure pain scores for intra-arterial lidocaine (IAL) and control. Supplementary Figure S5: Forest plot comparing WBC count for intra-arterial lidocaine (IAL) and control. Supplementary Figure S6: Forest plot comparing C-Reactive Protein (CRP) for intra-arterial lidocaine (IAL) and control. Supplementary Figure S7: Sensitivity analysis forest plot comparing 4-h post-procedure pain scores for intra-arterial lidocaine (IAL) and control. Supplementary Figure S8: Sensitivity analysis forest plot comparing maximum 24-h pain scores for intra-arterial lidocaine (IAL) and control.
