# Supplementary material for: Intra-arterial lidocaine as a pain management strategy for uterine artery embolisation: a systematic review and meta-analysis
Source: CVIR Endovasc. 2026 Sep 16;9:121. doi: 10.1186/s42155-026-00771-y (PMC13582736; doi:10.1186/s42155-026-00771-y)
Supplement: Supplementary file 1 [file 42155_2026_771_MOESM1_ESM.docx]

**Intra-Arterial Lidocaine as a Pain Management Strategy for Uterine Artery Embolization: A Systematic Review and Meta-Analysis**

**Supplementary Information**

**Affiliations**

Tarun Haridasan^1^, Abena Kena^1^, Larry Nguyen^1^, Kiat Tsong Tan^1, 2^

^1^ Michael G. DeGroote School of Medicine, McMaster University, 1280 Main Street West, Hamilton, Ontario L8S 4L8, Canada
^2^ Department of Medical Imaging, Niagara Health System, St. Catharines, Ontario L2S 0A9, Canada

**Author Email Addresses**

Tarun Haridasan: tarun.haridasan@medportal.ca

Abena Kena: abena.kena@medportal.ca

Larry Nguyen: larry.nguyen@medportal.ca

Kiat Tsong Tan: KiatTsong.Tan@niagarahealth.on.ca

**Corresponding Author**

Dr. Kiat Tsong Tan

Michael G. DeGroote School of Medicine

McMaster University

1280 Main Street West, Hamilton, Ontario L8S 4L8, Canada

Email: KiatTsong.Tan@niagarahealth.on.ca

Phone: (306) 715-7594

**Pain Scores**

**Supplementary Figure 1. Forest plot comparing 0-hour post-procedure pain scores for intra-arterial lidocaine (IAL) and control.** M-H, Mantel-Haenszel; IV, inverse variance; CI, confidence interval.

**Supplementary Figure 2. Forest plot comparing 4-hour post-procedure pain scores for intra-arterial lidocaine (IAL) and control.** M-H, Mantel-Haenszel; IV, inverse variance; CI, confidence interval.

**Supplementary Figure 3. Forest plot comparing 7-hour post-procedure pain scores for intra-arterial lidocaine (IAL) and control.** M-H, Mantel-Haenszel; IV, inverse variance; CI, confidence interval.

**Supplementary Figure 4. Forest plot comparing 24-hour post-procedure pain scores for intra-arterial lidocaine (IAL) and control.** M-H, Mantel-Haenszel; IV, inverse variance; CI, confidence interval.

**Inflammatory Markers**

**Supplementary Figure 5. Forest plot comparing WBC count for intra-arterial lidocaine (IAL) and control.** M-H, Mantel-Haenszel; IV, inverse variance; CI, confidence interval.

**Supplementary Figure 6. Forest plot comparing C-Reactive Protein (CRP) for intra-arterial lidocaine (IAL) and control.** M-H, Mantel-Haenszel; IV, inverse variance; CI, confidence interval.

**Sensitivity Analysis**

**Supplementary Figure 7. Sensitivity analysis forest plot comparing 4-hour post-procedure pain scores for intra-arterial lidocaine (IAL) and control.** Sensitivity analysis involved excluding the study by Cai et al., which included patients undergoing uterine artery embolization for cesarean scar pregnancy. M-H, Mantel-Haenszel; IV, inverse variance; CI, confidence interval.

**Supplementary Figure 8. Sensitivity analysis forest plot comparing maximum 24-hour pain scores for intra-arterial lidocaine (IAL) and control.** Sensitivity analysis involved excluding the study by Cai et al., which included patients undergoing uterine artery embolization for cesarean scar pregnancy. M-H, Mantel-Haenszel; IV, inverse variance; CI, confidence interval.
